# Obesity phenotypes and cardiovascular risk: From pathophysiology to clinical management

**DOI:** 10.1007/s11154-023-09813-5

**Published:** 2023-06-26

**Authors:** Alberto Preda, Federico Carbone, Amedeo Tirandi, Fabrizio Montecucco, Luca Liberale

**Affiliations:** 1 Vita-Salute San Raffaele University, Milan, Italy; 2 IRCCS Ospedale Policlinico San Martino Genoa – Italian Cardiovascular Network, Genoa, Italy; 3 Department of Internal Medicine, University of Genoa, 6 viale Benedetto XV, 16132 Genoa, Italy

**Keywords:** Obesity, Cardiovascular risk, Inflammation, Insulin resistance, Fat, Adipose tissue

## Abstract

Obesity epidemic reached the dimensions of a real global health crisis with more than one billion people worldwide living with obesity. Multiple obesity-related mechanisms cause structural, functional, humoral, and hemodynamic alterations with cardiovascular (CV) deleterious effects. A correct assessment of the cardiovascular risk in people with obesity is critical for reducing mortality and preserving quality of life. The correct identification of the obesity status remains difficult as recent evidence suggest that different phenotypes of obesity exist, each one associated with different degrees of CV risk. Diagnosis of obesity cannot depend only on anthropometric parameters but should include a precise assessment of the metabolic status. Recently, the World Heart Federation and World Obesity Federation provided an action plan for management of obesity-related CV risk and mortality, stressing for the instauration of comprehensive structured programs encompassing multidisciplinary teams. In this review we aim at providing an updated summary regarding the different obesity phenotypes, their specific effects on CV risk and differences in clinical management.

## Introduction to obesity and cardiovascular risk

Over the last 30 years, the epidemic of overweight and obesity has increased dramatically, reaching the dimension of a real global health crisis [1]. According to the data of the World Health Organization, more than 1 billion people worldwide are living with obesity (650 million adults, 340 million adolescents and 39 million children) accounting for about 2.8 million deaths every year [2]. Adipocytes secrete different hormones and peptides under several physiological and pathological conditions, known globally as adipokines and playing an important role in local and systemic regulation of energy homeostasis and inflammation [3–5]. Multiple obesity-related mechanisms are cause of structural, functional, humoral and hemodynamic alterations believed to underpin the development of CVD including atherothrombosis, atrial fibrillation (AF) and myocardial dysfunction [6–8]. Thus, a correct assessment of the cardiovascular (CV) risk in people with obesity is critical for reducing mortality and preserving quality of life in this class of patients. However, the correct identification of the obesity status is still tricky as recent evidence suggest that different phenotypes of obesity exist, each one associated with different degree of CV risk [9, 10]. Body mass index (BMI) has been longtime indicated as golden standard to assess adipose depots and the associated cardiovascular risk, but several limitations apply [8]. Considerable variations occur according with sex, age, and race/ethnicity. In the last decade, a shift toward a qualitative approach led to rephrase the paradigm of obesity into the concept of obesities [11]. With time, several other anthropometric measures have made their way alongside or replacing BMI: mainly waist circumference (WC) [12, 13] but also, waist-hip ratio (WHiR), waist to height ratio (WHtR), bioimpedance, 3D scanning and dual energy x-ray absorptiometry (DEXA). Such a paradigm shift takes into great account qualitative differences in adiposity as associated with different degrees of metabolic and atherogenic derangements and different responses to weight loss, lifestyle modification or medications. Recently, the World Heart Federation (WHF) and World Obesity Federation (WOF) provided an action plan for management of obesity-related CV risk and mortality, stressing for the institution of comprehensive structured programs encompassing multidisciplinary teams [14]. In this review we aim at providing an updated summary regarding the different obesity phenotypes, their specific effects on CV risk and differences in clinical management.

## The journey from BMI to visceral adiposity and obesity phenotypes

### The obesity paradox

Historically, the increase in adiposity depots expressed by BMI is linearly associated with growing CVD risk and mortality. Nevertheless, the first decade of this century saw the emergence of a mismatch between the awareness of excess body weight burden and its related metabolic consequences. The concept of ‘obesity paradox’ was born, and scientist stayed in this swamp for a decade further [15]. In several studies patients with obesity have indeed shown a better prognosis as compared with leaner ones [16]. Gruberg and co-workers firstly described this evidence in patients affected by coronary artery disease (CAD) undergoing percutaneous coronary intervention (PCI) [17]. Subsequently, numerous other conflicting data where published regarding the benefits of weight reduction in some high-risk CV conditions—heart failure (HF), atrial fibrillation (AF) or hypertension—as well as other non-CV conditions such as frailty, diabetes mellitus (DM), end-stage renal disease and chronic obstructive pulmonary disease [18, 19]. Notably, in patients affected by chronic HF, those losing more weight over time also showed higher mortality rate [20]. Numerous possible explanations to this phenomenon were provided. First, patients with obesity and CVD are on average younger and with better conserved systolic function than lean patients. Acute myocardial infarction (AMI) in patients with obesity has been found to be associated with less severe and complex CAD than in non-obese subjects [21]. Moreover, patients with obesity have higher levels of arterial pressure, thus they can be exposed to higher dosages of anti-ischemic and anti-remodeling medications. Nevertheless, the higher survival after AMI in this population was found to be independent of their younger age and more intensive medication treatment [22]. Other clinical features may in part explain the reduction of hospitalization time, as well as short- and long-term mortality [21, 23]. Different confounding factors (e.g. smoking, chronic illness, lung disease, cancer) as well as reverse causality were also pointed out as possible explanations for the OP. Indeed, the severity of the disease could strongly impact the weight loss trajectory. On the other hand, unintentional weight loss is often marked by relative reduction of muscle mass and peripheral fat, rather than central fat [24]. This phenomenon cannot be discriminate by the use of BMI [25]. The predictive role of WHR seems to be higher for CV risk stratification in those patients [26, 27]. In a recent study, WHR and WC better correlate with the severity of CAD in patients undergoing PCI while BMI only showed a low predictive value [28]. Markers of central fat should be considered better indicators of future risk in this context [29].

### “Adiposopathy” and “diabesity”

Adipocyte hypertrophy in visceral adipose tissue and ectopic fat accumulation leads to cellular dysfunction, metabolic abnormalities and endocrine disturbances [30]. Adipose tissue dysfunction also known as “adiposopathy” is a root cause of some of the most common metabolic diseases observed in clinical practice, including DM, hypertension and dyslipidemia [31]. While classically related to the visceral fat, growing evidence suggest a role for dysfunctional stimulation of the subcutaneous adipose tissue in obesity [32]. Metabolic consequences of adiposopathy have been traditionally clustered in the general term metabolic syndrome (MetS) accounting for central obesity, hyperglycemia, hypertriglyceridemia, low levels of HDL and hypertension [33]. Shift toward visceral adipose tissue distribution, ectopic fat deposition and inflammatory/adipokines dysregulation are now considered the central tenets of adiposopathy [34]. Hypertrophic adipocytes showed an unbalanced adipokines production, promoting insulin resistance (IR), inflammation, fatty liver, increased LDL-cholesterol, oxidative stress, endothelial dysfunction and pro-thrombotic state [35]. Among adipokines, leptin levels were shown to be directly proportional to obesity and body fat levels, while its counter-hormone adiponectin resulted reduced [36]. This imbalance is thought to enhance atherogenesis, fibrosis, hyperglycemia and inflammation [37, 38]. Chemerin, a newly characterized chemoattractant released by adipocytes, is gaining more and more attention as a potential MetS biomarker being related with adipogenesis, angiogenesis and glucose metabolism [39, 40]. In humans, chemerin positively correlates with adiposity [41, 42], independently from WC or BMI [42], and strongly predicts MetS development [43]. Adipocyte hypertrophy also leads to ischemic dysfunction and hypoxia-related signaling. The surrounding microenvironment then modifies its architecture. Inflammatory cells from both innate and adaptive immunity infiltrate the dysfunctional adipose tissue and activate inflammatory pathways that further sustain such pathophysiological processes. Among the other, the upstream mediator osteopontin (OPN) seems also to be strongly associated with adiposopathy and cardiometabolic consequences. Released by macrophage within dysfunctional adipose tissue, OPN sustains adipocyte and metabolic dysregulation in both experimental and clinical studies [44–46]. Lipolysis and insulin resistance finally characterize such a dysfunctional microenvironment and reach peripheral tissues (e.g., skeletal muscle and liver) [47, 48]. Especially within the skeletal muscle, decrease in GLUT-4 translocation reduces glucose uptake and facilitates glycogenolysis [49]. In the liver, FFAs promote gluconeogenesis and lipogenesis further increasing insulin levels. Again, within pancreas islets, FFAs exert lipotoxic effect on beta cells leading to reduced insulin secretion and a failure of compensation [50]. Since adiposity and DM are strictly related, the term “diabesity” was coined to describe the superadded effects of DM and obesity on CV risk [51].

## Obesity phenotypes

Pitfalls in the characterization of body fat distribution through the BMI and distinction of fat *vs.* lean tissue have provided a critical contribution to explain the non-unique subdivision of obesity phenotypes among studies. Based on current knowledge, “obesities” may be categorized across four groups: i) metabolic unhealthy normal weight (MUNW), ii) metabolically healthy overweight/obese (MHO), iii) metabolically unhealthy overweight/obese (MUO), and iv) sarcopenic obesity (SO) (Table 1). MHO and MUO are the most representative categories, including patients with a BMI > 25 kg/m [2] but very different metabolic profile [52] (Table 2). Alterations in body fat distribution is the key factor characterizing those two phenotypes. MUO encompasses the old features of MetS, which translates in a higher cardiometabolic risk [53]. In addition to age and higher WC, reduced subcutaneous fat and shift toward a visceral and dysfunctional/pro-inflammatory hypertrophic adipose tissue distribution characterize MUO. Impaired fat storage and ectopic visceral fat deposition in liver and skeletal muscle further characterize this prototypic phenotype of adiposopathy [54, 55]. Contrariwise, the healthier MHO phenotype is less common among European population, with a prevalence of 10–30% [56]. They are more often young, female, physically active people with a better nutritional status [57]. Although a definition of MHO is not standardized yet [58], this group would include people with high BMI and healthy metabolic profile: preserved insulin sensitivity, favorable lipid profile and low plasma levels of pro-inflammatory cytokines [59]. Nevertheless, even the alleged lower CV risk associated with MHO has been questioned [60]. Although CV risk did not differ from normal weight individuals, MHO had significantly higher risk to develop MetS over time and then increase by about 60% the chance of suffering major CV events in the MESA study [61, 62].

**Table 1:**
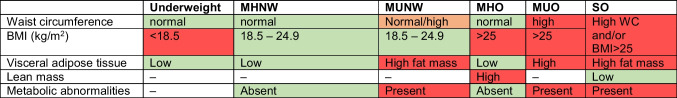
Summary of defining criteria of different obesity phenotypes

Normal values are in green, pathological ones are in red, and the intermediate ones in orange. Waist circumference categorized as normal (men < 102 cm and women < 88 cm) or high (men ≥ 102 cm and women ≥ 88 cm). Visceral adipose tissue and lean mass are a non-standardized measure actually. Metabolic abnormalities refer to the metabolic syndrome defining criteria

*BMI* body mass index, *MHNW* metabolically healthy normal weight, *MUNW* metabolically unhealthy normal weight, *MHO* metabolically healthy overweight/obese, *MUO* metabolically unhealthy overweight/obese, *SO* sarcopenic obese

**Table 2:** Over time development and controversies in definition of metabolically healthy/unhealthy overweight/obese

**Wildman et al*.*** [90]	**BioSHaRE-EU Healthy Obese Project** [56]		**Lavie et al*.*** [91]
	Less strict	Stricter	
IFG/IGT/T2DM	FPG > 126 mg/dL	FPG > 110 mg/dL	FPG > 100 mg/dL
BP ≥ 130/85 mmHg	BP > 140/90 mmHg	BP > 130/85 mmHg	BP > 130/85 mmHg
TAG > 150 mg/dL			
HDL < 40 (W) or < 35 (M) mg/dL			
WC > 88 cm (W), > 102 cm (M)			
HOMA-IR [92]			
hsCRP			
BMI > 25 or > 30 kg/m [2]	None (MHO) or ≥ 1 (MUO)		0–1 (MHO) or ≥ 1 (MUO)

*IFG* impaired fasting glucose, *IGT* impaired glucose tolerance, *T2DM* type 2 diabetes mellitus, *BP* blood pressure, *TAG* triglycerides, *HDL* high-density lipoprotein, *WC* waist circumference, *BMI* body mass index, *CAD* coronary artery disease, *HOMA-IR* homeostasis model assessment for insulin resistance, *hsCRP* high-sensitivity C-reactive protein, *MHO* metabolically healthy overweight/obese, *MUO* metabolically unhealthy overweight/obese

Similarly, a recent report from a UK biobank including > 380.000 people characterized MHO as at increased risk of HF (76%), respiratory diseases, all-cause mortality, and atherosclerotic CVD (20%) as compared to normal weight/MHO individuals [63]. Despite a lower baseline CV risk, MHO then develops atherosclerotic CVD risk factors earlier than lean individuals. Moreover, overweight itself is a non-negligible adverse factor that affects the natural history of several comorbidities such as respiratory, renal, and orthopedic ones [64, 65]. The MUNW group is another paradigm of the prevalent qualitative – rather than quantitative – relevance of adiposity (Table 3). They share similar CV risk factors [66] and metabolic alterations with traditionally patients with obesity, including chronic low-grade inflammation [67, 68]. MUNW has the highest rate of underdiagnoses among obesity phenotypes due to both the lack of consensus definition and the limited access to diagnostic tools for discriminating increased visceral adiposity and/or unbalanced fat/lean mass ratio [69, 70]. Its prevalence is estimated in high as 67% [71]. MUNW may or not be associated with changes in other anthropometric parameters, such as WC, WHiR, WHtR. The threshold of body fat mass applied in MUNW diagnosis varies among different studies, ranging from 19 to 32% for men and from 29 to 44% for women [72]. MUNW usually includes older and sedentary individuals [73] with generally a very low amount of gluteo-femoral fat mass compared with the visceral one [74]. Cardiometabolic risk associated with MUNW is high and high risk of CVD independently of elevated trunk fat mass as reported in lean women from Women’s Health Initiative Study [75]. MUO and MUNW phenotypes genetically differ: a variability in loci regulating food intake is reported in MUO, whereas genetic characterization of MUNW has highlighted a prevalence in genes regulating adipocyte differentiation, lipogenesis, and lipolysis (e.g. *IRS1, GRB14, PPARG, LYPLAL1)* [76, 77].

**Table 3:**
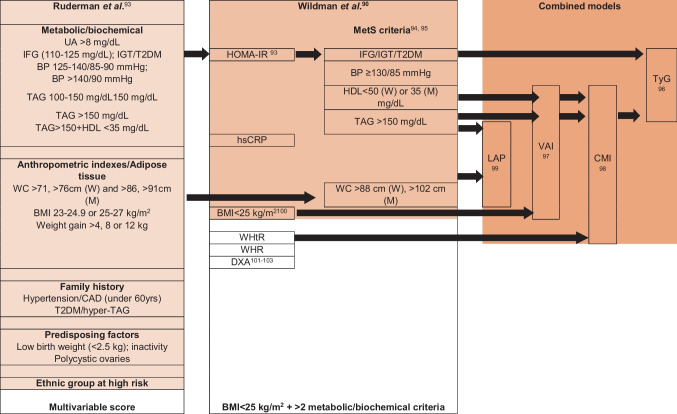
Over time development and controversies in definition of metabolically unhealthy normal weight

*UA* uric acid, *IFG* impaired fasting glucose, *IGT* impaired glucose tolerance, *T2DM* type 2 diabetes mellitus, *BP* blood pressure, *TAG* triglycerides, *HDL* high-density lipoprotein, *WC* waist circumference, *BMI* body mass index, *CAD* coronary artery disease, *HOMA-IR* homeostasis model assessment for insulin resistance, *WHtR* waist-to height ratio, *WHR* waist-to-hip-ratio, *DXA* dual-energy X-ray absorptiometry, *LAP* lipid accumulation product; *VAI* visceral adiposity index, *CMI* cardiometabolic index, *TyG* triglycerides-glucose index [90, 93–103]

As additional phenotype, SO is characterized by low skeletal muscle mass due to metabolic changes secondary to a sedentary lifestyle, adipose tissue derangements or chronic comorbidities [78]. Loss of skeletal muscle mass and function generally occurs with ageing and is commonly paralleled by relative or absolute body fat gain, favoring the potential development of SO. Adipose tissue has indeed a negative impact on muscle mass both directly through metabolic derangements (i.e. inflammation and IR) [79] and indirectly through increased prevalence of obesity-related chronic diseases with a negative impact on muscle metabolism (i.e., orthopedic disorders). Of interest, the skeletal muscle is now increasingly considered as an endocrine organ secreting a large number of factors, termed myokines, that favour the metabolic dialogue between the muscle and other organs, including the adipose tissue [80]. Although diagnostic criteria are variable among studies, SO is usually diagnosed when parameters of altered skeletal muscle strength coexist with altered body composition, in particular increased fat mass and reduced muscle mass [81]. Preclinical and clinical studies suggest the existence of a biological connection between IR, obesity and sarcopenia, mediated by the impaired function of the growth differentiation factor myostatin [82]. Such mediator, historically recognized among most important negative regulators of muscle mass, recently gains notoriety due to its role on glucose and fat metabolism including inhibition of insulin signaling, lipid oxidation and energy expenditure [83]. In addition to myostatin, sarcopenia and sarcopenic obesity are associated with a dysregulation of other myokines with important cardiometabolic functions, such as IL-6, FNDC5/irisin, fibroblast growth factor 21 or brain-derived neurotrophic factor, which play a critical role in skeletal muscle mass and function as well as metabolic homeostasis [84]. In SO, obesity and sarcopenia may therefore synergistically enhance each another with a vicious cycle facilitating weight gain and muscle loss through reduced mobility, dependency and disability [85]. As a consequence, such individuals show higher rate of adverse health consequences including falls and fractures, decreased mobility [86], poor quality of life and hospitalization [87] as compared to patients with isolated obesity or sarcopenia. Furthermore, systematic reviews and metanalysis report SO as a strongly predictor for all-cause mortality [88, 89].

## How does obesity affect the heart?

### Inflamm-aging and metaflammation

The term “inflamm-aging” merges two words “inflammation” and “aging” to describe the chronic, sterile, low-grade inflammation characterizing elderly individuals and playing fundamental roles in different age-dependent chronic diseases or conditions [5, 104–107]. Increased body fat composition and IR strongly associates with aging through several cellular and molecular mechanisms including cellular senescence, mitochondrial dysfunction, impaired autophagy and dysbiosis [108]. Moreover, the impaired crosstalk between adipocytes and the immune cells infiltrating the adipose tissue as well as the degeneration of self- and non-self-receptors is thought to contribute to the establishment of inflamm-aging itself [109]. Of interest, the innate immune response activates after food ingestion [110]. The so-called “postprandial inflammation” is part of the adaptive response to meals and causes the release of several pro-inflammatory mediators [111]. Therefore, the excess nutrients intake characterizing obesity associates with higher levels of inflammatory hormones (i.e. leptin) secreted by adipose tissue, leading to a metabolic reprogramming of immune cells, in particular macrophages, towards a pro-inflammatory phenotype. Such condition – known as “metaflammation” – synergistically works with accelerated inflammaging to create a dysregulated energetic environment, whose metabolic hallmarks are high levels of lipids, free fatty acids, glucose, and reactive oxygen species (ROS). Prolonged mitogenic signal induced by chronic hyperinsulinemia leads dysfunctional hypertrophic adipocytes to activate a post-mitotic cell cycle that initiate a senescent cell program. This process is associated to a pro-inflammatory secretome, which sustains and further contributes to low-grade chronic inflammation [112]. Macrophages and adipocytes demonstrate remarkable functional overlap, as both cell types secrete cytokines and can be activated by bacterial products (i.e. lipopolysaccharide) [113]. Furthermore, pre-adipocytes can transdifferentiate into macrophages. Of interest, whereas inflammation-resolving M2 macrophages dominate insulin-sensitive adipose tissue in the lean, pro-inflammatory M1 macrophages accumulate in parallel to adiposity in individuals with obesity, promoting inflammation and IR. Indeed, M1/M2 ratio indirectly correlates with both tissue-specific and whole-body insulin sensitivity [114]. Dysfunctional adipocytes induce M1 phenotype shifting by altering several intracellular pathways including IKK, JNK1, HIF and TLR signals against IL-4- and IL-13-mediated phosphorylation of STAT6 and expression of the lipid-sensing nuclear factors PPAR-γ and PPAR-δ [115, 116] M1 macrophages produce IL-1β, IL-6, TNFα and ROS further reducing insulin signaling in adipocytes. As a result, the number of M1 macrophages parallels the expansion of adipose tissue, exacerbating inflammation and IR. Many of those mediators may be clustered in the emerging concept of senescence-associated secretory patterns (SASP), increasingly considered leading driver of age-related disorders [117]. Among different molecules our research group has long time focused on the role of OPN – above described as upstream mediators of adipocyte dysfunction – is an interesting candidate bridge with cardiometabolic risk [118, 119]. Finally, gut microbiota also plays central roles in energetic homeostasis, as it can release inflammatory and anti-inflammatory products contributing to metaflammation [120]. Patients with obesity present a characteristically overgrowth of *Firmicutes* phyla (i.e. *Lactobacillus* and *Faecalibacterium*) and *Escherichia coli* against *Bacteroidetes* [121]. Such “obese microbiota” showed higher ability to extract calories from the diet [122] as well as being associated with increased gut permeability, leading to increased absorption of bacterial endotoxins [123]. The gut microbiota produces a wide variety of metabolites because of the anaerobic fermentation of undigested food [124]. Short-chain fatty acids (SCFAs) including acetate, propionate and butyrate are main metabolites of gut microbiota providing important anti-inflammatory effects. Studies showed that a reduction in the levels of SCFAs generate intestinal inflammation and foam cell formation, contributing to gut barrier disruption and favoring bacterial translocation including mobilization of lipopolysaccharides (LPS), trimethylamine N-oxide (TMAO) and phenylacetyl glutamine (PAGIn) which, in general circulation, induce systemic inflammation, macrophage activation and favor atherosclerosis [125].

### Cardiac fibrosis

The strong association between obesity and CVD directly involves the heart, independent of the atherosclerotic process. Several stress factors are involved in substantial changes at molecular, cellular, and interstitial levels in obese hearts including dysregulated activation of different neuro-hormonal systems, hyperinsulinemia and inflammation [126]. Cardiac cells respond to such an environment eliciting the hypertrophic growth response through secretion of cytokines, growth factors (GFs), vasoactive peptides, and hormones [127]. Although considered an adaptation mechanism, such response associates with cell death, fibrosis, and microvascular dysfunction. Cardiac fibrosis plays an important role in the pathogenesis of heart disease in patients with obesity causing impaired diastolic function, altered contraction, atrial and ventricular remodeling eventually leading to heart failure with preserved ejection fraction (HFpEF), atrial and ventricular tachyarrhythmias and increased incidence of sudden death [128]. Cardiac fibroblasts are the most abundant interstitial cells in myocardium and are responsible for the formation and preservation of the matrix network [129]. Cardiac fibroblasts can influence cardiac function through direct and indirect effects on cardiomyocytes [130]. While in young individuals, cardiac fibroblasts maintain quiescence exhibiting limited inflammatory or proliferative activity, in aging hearts cardiomyocyte loss parallels the expansion of the interstitium and increased collagen content due to activation of fibroblasts [131, 132]. Documentation of cardiac fibrosis in the isolated obesity is challenging considering its common association with conditions affecting the cardiac interstitium (such as hypertension and DM). Effects of the activation of the renin–angiotensin–aldosterone system (RAAS) is consistently noted in the fibrotic myocardium of these patients. Several cellular pathways are involved in the fibrogenic program [133]. The link between an overactive TGF-β cascade and cardiac fibrosis is well-established and mediated through effects involving Smad signaling [134, 135]. TGF- β stimulates different other GFs (i.e. epidermal GF, insulin-like GF-1, growth differentiation factor-11 and CTGF) involved in the inhibition of myofibroblast apoptosis leading to a vicious circle of sustained and progressive fibrotic response [136]. The altered adipokine balance also play a role in cardiac fibrosis and dysfunction. Impaired leptin/adiponectin ratio was implicated in the pathogenesis of cardiac remodeling in obesity and metabolic dysfunction being a marker of inflammation [137]. Elevated circulating leptin levels were associated with left ventricular hypertrophy and fibrosis [138, 139]. On the contrary, adiponectin exerts anti-fibrotic and anti-inflammatory effects on cardiac fibroblasts, presumably mediated by PPAR-α activation [140, 141]. OPN has been widely associated with cardiac remodeling in both experimental and clinical studies [142, 143].

Although not listed among adipokines, neprilysin is largely expressed on the surface of mature adipocytes in people with obesity [144]. This molecule degrades endogenous natriuretic peptides increasing renal sodium reabsorption, aldosterone secretion from the adrenal gland, cardiac inflammation and fibrosis. In subjects with obesity and HFpEF soluble neprilysin levels and its inhibition decreased ventricular overload and improved LA overfilling [145].

**Fig. 1:**
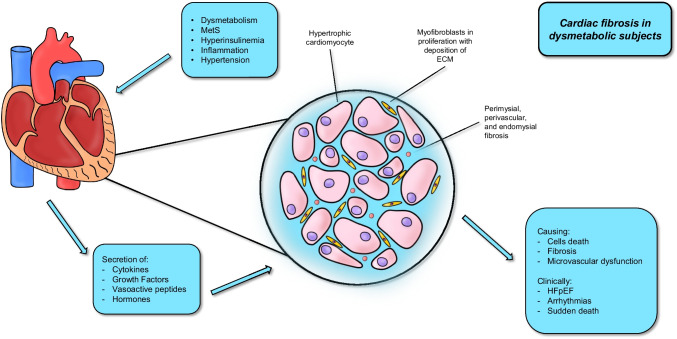
Cardiac fibrosis in dysmetabolic subjects. Patients with dysmetabolism are at higher risk of developing cardiac fibrosis. The long-term exposure to inflammatory, oxidative, and hyper-insulinemic environment causes the secretion of several molecules that concur in causing cardiac fibrosis. Microscopically, this process causes cells deaths, microvascular damages, and deposition of excessive extracellular matrix. Consequently, patients frequently experience heart failure, especially HFpEF, eventually arrhythmias, and even sudden death. HFpEF, heart failure with preserved ejection fraction; TGF-β, transforming growth factor beta; TSP-1, tronbospondin-1

Matricellular proteins are upregulated in remodeled hearts and regulate inflammatory, fibrotic and angiogenic responses [146]. Thrombospondins (TSP), tenascins, Cilp-1, secreted protein acidic and rich in cysteine (SPARC), osteopontin and members of the CCN family are involved in a variety of cardiac pathophysiologic conditions such as MI, cardiac hypertrophy, aging, diabetic cardiomyopathy and valvular disease. TSP-1 is the best-characterized matricellular protein in obesity, DM and MetS and it is potently induced by hyperglycemia [147]. The role of TSP-1 in cardiac remodeling was largely explored in clinical and preclinical studies, confirming its regulatory effect in fibrotic response of injured myocardium, deposition of collagen and angiogenesis [148-150]. Accordingly, such mediator is increasingly seen as a potential target for novel drugs in this context (Fig. 1).

### Ectopic adipose tissue

Obesity-related vascular dysfunction is not only characterized by increased collagen deposition within the vascular wall and progressive arterial thickening but also by perivascular fat accumulation and inflammatory infiltrate [151]. Perivascular adipose tissue (PVAT) is located around most large blood vessels close to the vasculature and direct contact with the adventitia, providing mechanical protection and regulation of blood vessel tone via paracrine and vasocrine pathways [152, 153]. PVAT’s phenotype is heterogeneous and strongly location-dependent [154, 155]. In lean individuals, PVAT is mostly thermogenic brown and beige, located in the cervical, supraclavicular, axillary, paraspinal, renal and epicardial regions [156, 157]. Instead, the abdominal aorta and mesenteric vasculature are surrounded by white adipocytes, also found in visceral and subcutaneous adipose depots [158]. Functional PVAT secrets a number of adipokines (i.e. adiponectin and angiotensin 1–7) with antithrombotic and vasodilating effect on the vasculature [159, 160]. Moreover, PVAT is populated with different immune cells important for vascular homeostasis (i.e. regulatory T-cells) [161]. Obesity induces changes in the vasoactive factors in which the beneficial paracrine effect of PVAT is shifted to a pro-oxidant, pro-inflammatory, contractile and trophic environment [162]. Furthermore, the dysfunctional PVAT promote endothelial dysfunction, atherogenesis, vascular IR, impaired relaxation, and vascular stiffness. Quite different from PVAT, the interest to the epicardial one (EAT) has grown rapidly in the past decade after the discovery of its role in physiological and pathological modulation of coronary homeostasis. EAT is located on the surface of the myocardium in direct contact with coronaries and accounts for ≈5% to 20% of the heart weight [163]. Age, WC, ethnicity, and cardiac mass are independent determinants of EAT volume [164]. Of interest, EAT volume is a known risk factor for CAD, HFpEF and AF [165]. Specifically, EAT thickness has been correlated with the presence of high-risk/unstable coronary plaques [166] and coronary microvascular impairment [167, 168]. Similarly to PVAT, EAT releases factors (i.e. adiponectin, leptin omentin-1, nitric oxide, palmitic acid methyl ester prostacyclin) and cytokines that affect both vascular and myocardial homeostasis through paracrine and vasocrine pathways [169]. Recent studies focused on the role of EAT-released exosomes, through which EAT carries lipids, proteins, ribonucleic acids (RNAs), and microRNAs, facilitating intercellular signaling. According to these studies, EAT’s exosomes may be implicated in a number of CVD such as MI, adverse cardiac remodeling and atrial fibrillation (AF) and are currently investigated for their potential role in modulation of myocardium healing [170, 171].

Although not close to myocardial tissue, ectopic fat accumulation in the liver, skeletal muscle, and kidney belong to the central tenets of adiposopathy [172]. Macrovesicular steatosis involving more than 5% of hepatocytes is considered the cut-off point triggering a multiple-hit cascade is mainly characterized by lipotoxicity, but would also include mitochondrial dysfunction, endoplasmic reticulum stress, hypoxia. Those mechanisms would include cytokine unbalance, hypothalamic signaling modifications and changes in microbiota. Although far from myocardial tissue, non-alcoholic fatty liver disease has been associated with right ventricular dysfunction and right bundle branch block, AF and QTc prolongation [173]. Although less is known about other ectopic fat depots, increasing data are describing those within skeletal muscle. They are highly expressed in diabetic patients and associated with cardiovascular risk and poor outcome after cardiovascular events [174]. Similarly, peri-renal fat has been demonstrated an index of sub-clinical atherosclerosis.

## Obesity phenotypes and cardiovascular risk

**Fig. 2:**
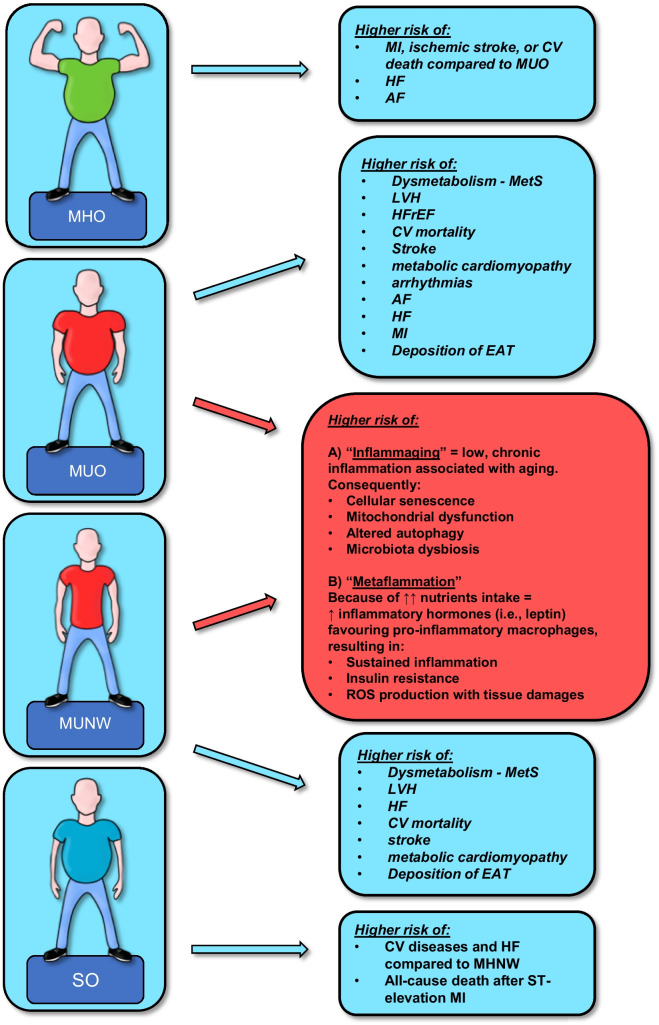
Obesity phenotypes and cardiovascular risk. This figure summarizes the close relationship between the different obesity phenotypes and the CV risk. AF, atrial fibrillation; CV, cardiovascular; EAT, epicardial adipose tissue; HF, heart failure; LVH, left ventricular hypertrophy; MetS, metabolic syndrome; MHO, metabolically healthy obese; MI, myocardial infarction; MUNW, metabolically unhealthy normal weight; MUO, metabolically unhealthy obese; ROS, reactive oxygen species; SO, sarcopenic obese

Obesity phenotypes have been shown to impact on CV diseases differently (Table 4 and Fig. 2). For coronary vascular and microvascular disease risk increases in MUO is proportional to the number of MetS defining criteria (hypertension, dyslipidemia, glucose intolerance and the degree of WC) [175, 176]. Direct negative effects of energetic dysmetabolism related to MUO and NUNW on cardiac structure are diverse and fall within the broad family of metabolic cardiomyopathies [177]. The hallmark of this condition is the development of left ventricle hypertrophy (LVH), independently related to the predominance of obesity, hypertension, and diabetes [178, 179]. The pathway from LVH to overt HF is complex and still partially unexplored, despite LVH being clearly recognized as an independent predictor of CV mortality [180], stroke, and renal outcomes [181]. Increased left ventricle stiffness and mass impairs the relaxation phase of the cardiac cycle leading to diastolic dysfunction, potentially leading to HF with preserved ejection fraction (HFpEF) [182]. To be noted, HF with reduced ejection fraction (HFrEF) is reported less frequently in patients with MUO and MUNW, and mostly associates with acute CV events (e.g., acute MI) [183]. Such negative structural and energetic remodeling is—together with inflammation and neuro-hormonal activation—a well-established substrate for arrhythmias [184]. In MUO, cardiac arrhythmias are frequent and precipitated by several co-factors including hypoxia, hypercapnia, electrolyte imbalances due to diuretic therapy, CAD and obstructive sleep apnea [185]. AF is the most common sustained cardiac arrhythmia diagnosed in individuals with obesity being an important determinant of stroke, HF, MI, dementia, and death in such population [186]. Of interest, positive correlations were found between the cumulative metabolic affliction and the risk of incident AF [187]. Of paramount, DM and hypertension are well-known independent risk factor of AF as well as criteria of the CHA2DS2-VASc-score [188]. As for the relationship between elevated TG and the risk of AF, reports remain controversial. While the Multi-Ethnic Study of Atherosclerosis (MESA) and the Framingham Heart Study (FHS) reported an association between hypertriglyceridemia and AF [189], this was not confirmed by the Niigata Preventive Medicine Study and by post-hoc analysis from the ARIC study [187, 190]. Obesity has been identified as the most common nonischemic cause of SCD [191]. Indeed, its association with SCD is well established [192] and every 5-unit increment in BMI indeed confers a 16% higher risk of SCD [193]. Cardiac fibrosis due to LVH, QRS fragmentation, QT prolongation, premature ventricular complexes, autonomic imbalance and increased EAT [194, 195] may explain the greater risk of ventricular tachycardia/ventricular fibrillation in such population [196].

**Table 4:** Summary of studies linking cardiometabolic disease with different obesity phenotypes

	**MUNW**	**MHO**	**MUO**	**SO**
MetS	–	↑ risk insulin resistance ↑ risk hyper-TAG ↑ risk low HDL ↑ risk hypertension *vs.* normal weight lean [217]	–	
Atherosclerosis	↑ vascular inflammation [203] ↑ PWV ↑ soft plaques [201] ↑ CACS ^218^*vs. *normal weight lean	↑ peripheral microvascular dysfunction (PMID: 28,275,071) ↑ cIMT [219, 220] ↑ CACS [218] *vs.* normal weight lean ↑ cIMT [221] *vs.* MUNW < 60y old	↑ peripheral microvascular dysfunction [222] ↑ cIMT [220] ↑ CACS [218] *vs.* normal weight lean ↑ cIMT [221] *vs.* MUNW < 60y old	↑ arterial stiffness [223] ↑ CACS [224] *vs.* non-sarcopenic ↑ cIMT [225] *vs.* non-sarcopenic elderly
HF	↑ LVsD ↑ LVdD [226, 227] ↑ risk [228] *vs.* normal weight lean ↑ risk [183] *vs.* normal weight lean post-menopausal woman ↑ LVH [178] *vs.* MHO	↑ risk [199, 229, 230] *vs.* normal weight lean ↑ LVdD [231] *vs.* MUNW ↑ risk [230] over time = risk than normal weight lean in post-menopausal woman (PMID: 33775111)	↑ risk [183] *vs.* normal weight lean post-menopausal woman ↑ LVH [178, 179, 232, 233] *vs.* MHO	↓ CRF [216, 234] *vs.* non-sarcopenic HFrEF
AF	↑ risk [228] *vs.* normal weight lean	↑ risk [186, 199, 229, 235] *vs.* normal weight lean ↓ risk [236] *vs.* MUO	↑ risk [186] *vs.* normal weight lean	↑ risk [225] *vs.* non-sarcopenic elderly
CV events/mortality	↑ risk [204, 230, 237] *vs.* normal weight lean ↑ risk [238] *vs.* obese (MHO/MUO)	↑ risk [199, 229, 237, 239, 240] *vs.* normal weight lean ↑ risk [206, 241] *vs.* MUNW	↑ risk [204, 230, 242] *vs.* normal weight lean	↑ risk [216, 243, 244] *vs.* non-sarcopenic HF and elderly ↑ risk [213] *vs.* non-sarcopenic after STEMI ↑ MI risk *vs.* non-sarcopenic elderly

Waist circumference categorized as normal (men < 102 cm and women < 88 cm) or high (men ≥ 102 cm and women ≥ 88 cm). Visceral adipose tissue and lean mass are non-standardized measures. Metabolic abnormalities refer to the metabolic syndrome defining criteria

*BMI* body mass index, *MONW* metabolically obese normal weight, *NOW* normal weight obese, *MHO* metabolically healthy obese, *MO* metabolically obese, *SO* sarcopenic obese

Few studies explored and compared the different mechanisms involved in the development of CV disease (CVD) in MHO and MUNW with respect to MUO and metabolically healthy individuals. Several studies suggested MHO as a pre-MUO condition with an intermediate risk of CVD between MUO and the healthy phenotype [197, 198]. However, this relationship may vary depending on the definition of MHO, the lack of adjustment for some confounding factors such as age, sex or a history of smoking, and the lack of separate analyses of the different subtypes of incident CV events. Individuals with MHO do not appear to carry a higher risk of MI, ischemic stroke, or CV death than healthy individuals. On the opposite, they show an increased risk HF and AF [199]. Similarly, in a nationwide analysis conducted in South Korea, Lee et al. reported a non-increased risk of ischemic stroke in MHO individuals [200].

On the contrary, MUNW is historically defined as a “fat mass disease” due to its higher risk of developing MetS and CVD despite normal weight [68]. Several large studies suggested the absence of correlation between normal weight and unhealthy status in patients with CV events, pointing out the possible role of other risk factors. MUNW individuals carry a higher incidence of subclinical atherosclerosis assessed by coronary computed tomography angiography as compared with healthy individuals [201]. Moreover, MUNW associates with soft atherosclerotic plaques [202] and subclinical vascular inflammation [203], known predictors of plaque rupture and ischemic events. The characterization of CV risk in such patients is far from being yet compete. Few clinical studies explored the incidence of CVD in this subgroup of obesity, reporting an increased risk of myocardial infarction in Chines [204] and Mexican American [205] populations. Of interest, a single study evaluated the incidence of HF in MUNW compared with MHO so far, reporting a twofold risk over 6 years [206]. Regarding AF, MUNW carries twofold increased risk as compared with healthy people or to MHO/MUO individuals [207].

Sarcopenia may promote atherogenesis due to relative fat mass increase in response to loss of muscle mass and replacement of myocytes by adipocytes. Hence, an even greater effect on CVD is expected for such a derangement with respect to obesity or sarcopenia alone [208]. Despite evidence on the relationships between SO and cardiovascular risk factors, its association with CVD is far from being clarified [209]. Cross-sectional studies have often yielded inconsistent results while prospective studies reported higher CV events in SO groups compared with the normal body composition groups only when SO was defined by using grip strength and WC criteria [86, 210]. In the Cardiovascular Health Study, a large prospective study of community-dwelling older men and women, SO based on WC and muscle strength was associated with the highest risk of CVD and HF over 8 years as compared with healthy subjects [211]. Few studies reported also higher incidence of myocardial infarction and AF, particularly in elderly [212]. Furthermore, patients with SO showed poor prognosis after STEMI, characterized by increased rate of all-cause death, MI, ischemic stroke, hospitalization for HF and unplanned revascularization [213]. The role of body composition in the development and progression of HF has recently received intense scrutiny [214]. In fact, in addition to cardiac dysfunction patients with HF also present abnormalities in body composition such as sarcopenia, SO and cachexia [215] with direct negative impact on their quality of life and survival. The FRAGILE-HF trial reported an high predictive role of SO in predicting mortality in adults with HF [216]. However, the lack of universally recognized diagnostic criteria remained a non-negligible factor which affects patient identification, reliable assessment of SO prevalence and outcomes. In 2022, the European Society for Clinical Nutrition and Metabolism (ESPEN) and the European Association for the Study of Obesity (EASO) provided the first consensus on SO definition, screening, diagnosis and staging [81]. Such consensus will help to uniform the selection criteria of SO patients in future studies.

## Therapeutic management of obesity phenotypes

Preliminary results suggest that the different obesity phenotypes also have different responses to weight loss interventions, including diets, medications, devices, and surgery [245]. Yet, by now no randomized controlled trials on obesity treatment compared cardiometabolic outcomes among individuals with different obesity phenotypes. However, numerous studies support the need for a stratification effort in relation to the type of obesity. Weight reduction approaches are initially based on incremented on physical activity implementation and dietary strategies. In patients living with obesity, regular physical activity and aerobic exercise provide a moderately reduction of risk factors for CAD, including body fat and body mass, blood pressure, triglycerides, and improved lipoprotein profile. Furthermore, physical activity improved insulin sensitivity and endothelial function regardless of weight loss. As a result, regular physical activity associates with a sensible improvement of obesity-associated complications including CAd [246]. As for the diet, despite the scientific soundness of energy restriction approaches, the evidence shows only modest effects with high individual differences and short duration. The Mediterranean dietary pattern has been widely recognized for its protective effects on obesity, CVD and DM in addition to decreasing all-cause mortality [247]. In MUO phenotype, weight loss is the cornerstone of the clinical management. Body weight reduction together with a low glycemic index diet have several beneficial effects on serum glucose, LDL and blood pressure improving CVD risk [248]. In patients with MHO weight loss strategies should be recommended to preserve cardiometabolic risk profile and avoid MHO/MUO conversion. Several studies highlighted the importance of improving fat oxidation in patients with MHO to prevent MetS/DM [249]. Suitable approaches include increased aerobic activity, Mediterranean diet, and supplementation with catechins, capsaicin, or L-carnitin [250, 251]. Regarding SO, both dietary interventions and regular exercise are reccomended [252]. Aerobic activity, resistance training and their combination increase muscle protein synthesis in older adults despite age-related decreases in anabolic signaling [253]. Furthermore, physical activity leads to the recruitment of muscle satellite cells located between myofibers and their surrounding basal lamina [254] and downregulation of inflammatory biomarker [255]. SO patients should be advised to follow a hypocaloric high-protein diet (1.2–1.4 g/kg body weight reference/day) to preserve their muscle mass [256]. On the opposite, significant weight loss is not recommended for individuals with MUNW. These individuals have less fat mass than other phenotypes, therefore, therapeutic strategies should focus on improving metabolic health and their effects on different adipose tissue compartments and on lipid accumulation in the liver. As an example, the Mediterranean diet reduces the risk of CV events by about 30%, compared with a control diet, despite having little effect on bodyweight [257]. Anti-obesity drugs have historically faced multiple issues relating to study design, premature termination due to safety issues or failure to show CV benefit [258]. Furthermore, there is no evidence on obesity phenotype‐specific effects of such medications to date. Metabolic/bariatric surgery remains the most effective strategy to accomplish a significant (≥ 30%) and durable (at ≥ 5 years) weight loss leading to reduced all-cause and CV mortality and lower incidence of several CVD [259]. However, this approach remains strictly recommended only for patients with complicated severe obesity.

## Conclusions

Guidelines from major European and American Societies highlight the importance of effective diagnosis and treatment of obesity in preventing CVD in clinical practice [8, 260, 261]. Obesity diagnosis may not be as simple as previously thought. Specifically, it cannot depend only on anthropometric parameters but should include a precise assessment of the metabolic status. Under this point of view different phenotypes of obesity have been proposed each one with specific effects on the CV system and with different responses to anti-obesity interventions. The current lack of standardized definitions reflects on a general paucity of experimental evidence impacting on the daily ability to provide personalized prescriptions to patients living with obesity. Such a complexity requires a multidisciplinary approach including specialists in obesity medicine, internal medicine, cardiology, psychology, as well as dieticians, family doctors, and bariatric surgeons. Accordingly, the therapeutic management of adiposopathy and its CV sequelae should be based on combination approaches encompassing surgery, pharmacotherapy, and lifestyle interventions.
